# Usefulness of serum interleukin-18 in predicting cardiovascular mortality in patients with chronic kidney disease – systems and clinical approach

**DOI:** 10.1038/srep18332

**Published:** 2015-12-16

**Authors:** Dorota Formanowicz, Maria Wanic-Kossowska, Elżbieta Pawliczak, Marcin Radom, Piotr Formanowicz

**Affiliations:** 1 Department of Clinical Biochemistry and Laboratory Medicine, Poznan University of Medical Sciences, Grunwaldzka 6, 60-780 Poznan, Poland; 2 Department of Nephrology, Transplantology and Internal Medicine, Poznan University of Medical Sciences, Przybyszewskiego 49, 60-355 Poznan, Poland; 3 Institute of Computing Science, Poznan University of Technology, Piotrowo 2, 60-965 Poznan, Poland; 4 Institute of Bioorganic Chemistry, Polish Academy of Sciences, Noskowskiego 12/14, 61-704 Poznan, Poland

## Abstract

The aim of this study was to check if serum interleukin-18 (IL-18) predicts 2-year cardiovascular mortality in patients at various stages of chronic kidney disease (CKD) and history of acute myocardial infarction (AMI) within the previous year. Diabetes mellitus was one of the key factors of exclusion. It was found that an increase in serum concentration of IL-18 above the cut-off point (1584.5 pg/mL) was characterized by 20.63-fold higher risk of cardiovascular deaths among studied patients. IL-18 serum concentration was found to be superior to the well-known cardiovascular risk parameters, like high sensitivity C-reactive protein (hsCRP), carotid intima media thickness (CIMT), glomerular filtration rate, albumins, ferritin, N-terminal prohormone of brain natriuretic peptide (NT-proBNP) in prognosis of cardiovascular mortality. The best predictive for IL-18 were 4 variables, such as CIMT, NT-proBNP, albumins and hsCRP, as they predicted its concentration at 89.5%. Concluding, IL-18 seems to be important indicator and predictor of cardiovascular death in two-year follow-up among non-diabetic patients suffering from CKD, with history of AMI in the previous year. The importance of IL-18 in the process of atherosclerotic plaque formation has been confirmed by systems analysis based on a formal model expressed in the language of Petri nets theory.

It is easy to realize that despite the enormous development in science, technology and medicine there are still many diseases difficult to treat. This is most likely due to the fact that they are the result of very complex processes occurring in the human body. Obviously, the complexity of these processes makes them extremely hard to understand their nature, but such understanding seems to be necessary for the development of effective prevention and treatment methods. Hence, there is a need for new approaches to study such processes and among them the systems approach is the most promising one.

There are a lot of diseases which should be investigated in this way; atherosclerosis, the dominant cause of cardiovascular diseases, being one of them, is a key example. For many decades researchers and clinicians have been working together to explore the nature of this disorder and to develop new drugs. Indeed, new theories, new phenomena and medicines have been developed leading to decline in death rate of cardiovascular diseases. However, the prevalence of atherosclerosis remains still very high. Several major risk factors have been identified, but to the present day we have failed to fully understand the pathogenesis of this complex process in which many important signaling pathways are disrupted. This issue becomes even more difficult when there is another chronic disease such as chronic kidney disease (CKD), which is very often a consequence of arteriosclerosis and/or accompanies it.

The incidence and severity of cardiovascular complications among patients with CKD is disproportionate compared to the known traditional atherosclerosis risk factors profile. This is partly due to the larger number of these factors and various phenomena that are accelerated in CKD^1^. An additional problem is the subsequent accumulation of modified proteins and toxins which are not cleared properly with the progression of renal dysfunction^2^. In the light of the influence of these factors, the composition of atherosclerotic plaques in CKD is much different compared to those without CKD^3^,^4^. Such composition contributes to the high complication rate in CKD patients, since the coronary plaques are dynamic structures reflecting a state of micro-inflammation, Th1/Th2 dysregulation, with a number of intermediate steps of which the final outcome (rupture) depends, among others, on the equilibrium between proteolytic activity and protein synthesis.

The complexity of atherosclerosis makes it particularly difficult to comprehend; thereby prediction of its clinical consequences requires sophisticated markers that would be involved in many signaling pathways. In order to determinate such markers a deep understanding of the atherogenesis is crucial. As previously mentioned, to study this phenomenon, a systems approach seems to be best suited. The basis of systems analysis is a formal model of the studied system. It can be expressed in languages of various branches of mathematics. In our study we have developed and analyzed Petri net based model of the process of IL-18 involvement in the formation of atherosclerotic plaque and the contribution of kidney disease in this phenomenon. The analysis of the model indicated that IL-18, due to its pleiotropic properties, is essential for many signaling pathways, what makes it an important player in atherosclerosis process and very attractive target for researchers.

IL-18, a unique cytokine that has been analyzed in this study, can initiate a cascade of pro-inflammatory cytokines and stimulate Th1 or Th2 response depending on cytokine milieu^5^. These two processes are crucial for accelerated atherosclerosis triggered by both the activation of immunological response, the retention of circulating cytokines, advanced glycation products and pro-oxidants^6^, which contribute to the pro-inflammatory state when renal function declines. IL-18, originally identified as a factor that induces synthesis of interferon-γ, was found to be highly expressed in human coronary plaques and to be responsible for their destabilization^7^. In addition, in one study it was confirmed that young and middle-aged patients with a recent acute myocardial infarction (AMI) have higher IL-18 concentration in serum than age- and sex-matched control subjects, showing that concentration of this cytokine is associated with severity of coronary atherosclerosis^8^. Although, there is no doubt that there is a close relationship between IL-18 and accelerated atherosclerosis in CKD, only in few studies this cytokine was evaluated among patients suffering from CKD^9^,^10^,^11^. In the face of unfavorable statistics in-depth understanding of the factors and finding markers underlying poor outcomes in patients with CKD is crucial.

The aim of this study was to investigate whether and how IL-18 concentration in serum is prospectively associated with cardiovascular deaths in the 2-year follow-up in patients with various stages of CKD and the history of AMI in the previous year, but without diabetes mellitus. For this purpose systems and clinical approach have been used.

## Results

### Systems approach

**Figure 1:**
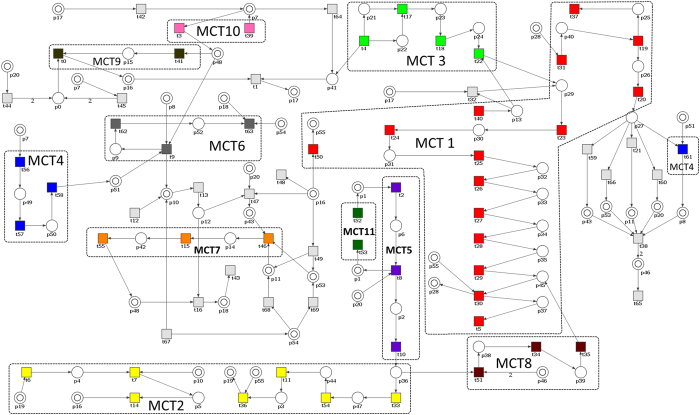
Petri net based model of the involvement of IL-18 in the formation of atherosclerotic plaque influenced by CKD (Some places, shown as double circles, are identically named but are presented as different nodes of the net. They represent the same passive components of the modeled system (i.e. all nodes with the same name represent the same place) but are presented as individual nodes in order to improve the readability of the model).

To show the importance of IL-18 in the signaling pathways of atherosclerosis that underlies cardiovascular disease, a formal model of the involvement of this cytokine in the formation of atherosclerotic plaque and its rupture in patients suffering from CKD, has been developed. The model, expressed in the language of Petri nets theory, which has been developed on the basis on available data from the literature^12^,^13^,^14^ and experience of our experts on this topic, is shown in Fig. 1. It consists of 56 places (*p*) and 70 transitions (*t*). There are 223 minimal t-invariants (i.e. t-invariants which are not linear combinations of other t-invariants) and no p-invariants. Moreover, there are 11 non-trivial MCT-sets (those ones which contain more than one transition). The lists of transitions and places with their biological meanings are shown in Tables A1 and A2, respectively, in Appendix while the biological interpretation of MCT-sets is provided in Table 1. These sets are also marked in Fig. 1. Of particular importance is transition *t*_67_ (CKD/hemodialysis) which is involved in 164 of the 223 minimal t-invariants, what means that it is an important player in the analyzed process and affects many signaling pathways. The analysis of the calculated t-invariants revealed that three transitions, i.e. *t*_43_ (atherosclerotic plaque formation), *t*_1_ (formation of the IL-18–IL-18R complex) and *t*_67_ (CKD/hemodialysis) may be found very often (n = 51) together in one t-invariant. Of these 51 minimal t-invariants of particular interest is the t-invariant no. 68 (*x*_68_), containing 45 of all of 70 transitions. It includes signaling pathways that are critical to the process tested. Among them are such paths/processes as: the myeloid differentiation primary response 88 (MyD88)-dependent signaling pathway, activation of inflammation via lipopolysaccharide (LPS) and its binding protein (LBP) mediated by a pair of sorting and signaling adaptor proteins, JAK/STAT (Janus kinase/signal transducer and activator of transcription) pathway stimulated by interferon gamma (INF-γ) and formation of an active tumor-necrosis factor receptor 1 (TNFR1) signaling complex. On the other hand, in 32 of 223 t-invariants were 3 transitions (*t*_1_, *t*_63_, *t*_67_) whose presence indicates that there is a close relationship between IL-18, CKD and atherosclerotic plaque rupture and cardiovascular event. On the basis of these results it can be concluded that IL-18 has a significant impact on the signaling pathways associated with atherosclerosis and its clinical consequences in patients with CKD.

### Clinical approach

#### Comparisons of the CKD patients and HV participants

All analyzed CKD patients (n = 126) met inclusion criteria for this study and were not excluded in the next stages of this study. They formed three groups (CKD1-2, CKD3-4 and CKD5d) differing in the stages of CKD progression. All of these patients were matched by the number of subjects and gender. For comparison, a reference group (HV) was created and matched to CKD patients on age and gender. Groups were dominated by middle-aged or older subjects; almost 60% of persons in each group were males. Patients classified to CKD1-2 and CKD3-4 groups showed mostly overweight, contrary to CKD5d and HV groups characterized by normal BMI.

There were significant differences in medications between the CKD groups. Although, patients were treated by recommended groups of drugs (angiotensin-converting enzyme inhibitors (ACEI), non-steroidal anti-inflammatory drugs (NSAID), beta-blockers and statins), not all of them received all of these medications. The greatest differences in this respect showed CKD5d patients.

Along with the progression of CKD, tendency to anemia, together with a decrease in serum iron and an increase in serum ferritin have been observed. Moreover, CKD patients were obviously more increasingly prone to have disturbances of phosphate metabolism, i.e. increased concentrations of PO_4_^3-^ and iPTH. Moreover, total protein concentration in serum was slightly decreased in CKD. With the deterioration of renal function lipid profile has changed, and most of studied patients showed dyslipidemia despite statins therapy.

Moreover, CKD patients showed elevated hsCRP levels. Furthermore, as the deterioration of renal function, significant gradual increase of NT-proBNP in human serum was observed. CIMT was significantly higher in all groups of CKD comparing to HV. Additionally, patients with CKD and progressive renal dysfunction showed higher levels of serum IL-18 compared to the HV ones. The baseline characteristics of the study population have been presented in Table 2.

#### Follow-up CV deaths

During 2-year follow-up 33 patients reach the endpoint (died because of cardiovascular-related events) because of fatal AMI (19 persons), fatal acute ischemic stroke (5 persons), sudden or unexpected death (9 persons). Moreover, 5 patients had another non-fatal AMI. The CV-related mortality (33 persons) was 26.19%; however, most CV-related deaths (14 persons) were recorded in CKD5d group (33.33% mortality).

##### ROC curve analyses

**Figure 2:**
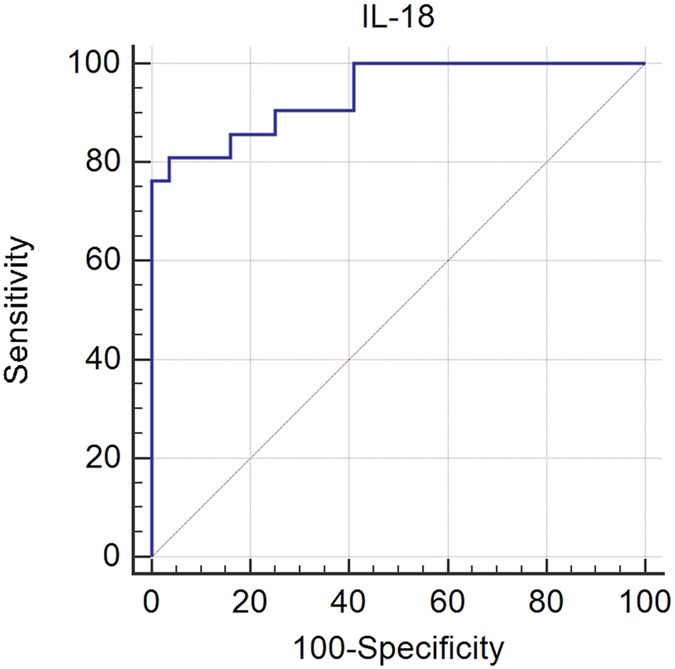
The receiver operating characteristic (ROC) curve for IL-18 in the serum indicating the optimal cut-off value of 1584.5pg/mL (sensitivity 81%, specificity 96.4%) that allows predicting CV-related mortality in post-myocardial infarction, non-diabetic patients with CKD.

The diagnostic utility of IL-18 was determined by means of the ROC curve analysis. On its basis the cut-off value for serum IL-18 above 1584.5 pg/mL has been determined. It allowed stratifying all CKD patients into 2 groups: CKD-A (IL-18 ≤ 1584.5 pg/mL) and CKD-B (IL-18 > 1584.5 pg/mL) with 81% sensitivity (95% confidence interval (CI) 58.1–94.6) and 96.4% specificity (95%CI 87.7–99.6). The area under the ROC curve (AUC) was equal to 0.94 (95%CI 0.86–0.98), *P* < 0.0001, Youden’s index = 0.77, see Fig. 2.

##### Characteristics of CKD-A and CKD-B groups

In the comparative analyses between the CKD-A (n = 91) and CKD-B (n = 35) groups significant differences in the eGFR, albumins, CIMT and NT-proBNP were found. In addition, it was disclosed that the vast majority of the CKD-B group created dialyzed patients (CKD5d group (n = 19)). In addition, their HD vintage was statistically significantly higher in comparison with CKD5d patients who were classified into CKD-A. On the other hand, in CKD-A there were most patients in the early stages of CKD (n = 40). Moreover, significant differences were observed in gender distribution. Clinical and biochemical characteristics of CKD groups divided into two groups: CKD-A and CKD-B has been presented in Table 3.

##### Survival analyses

**Figure 3:**
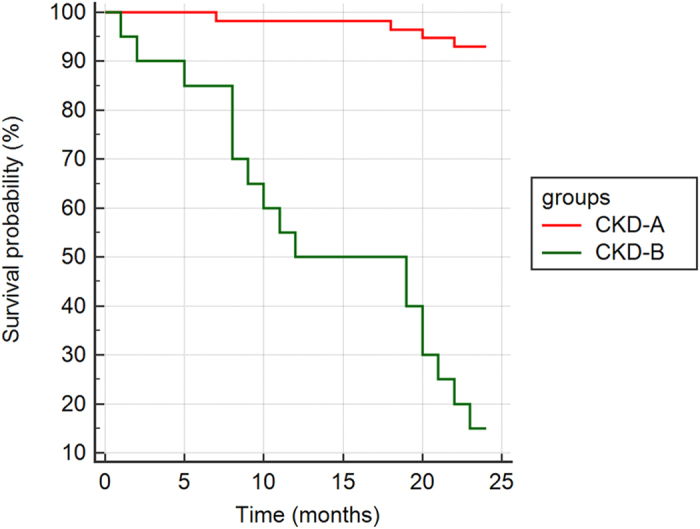
Kaplan-Meier survival curves of CV-related mortality in post-myocardial infarction patients with CKD divided into two groups (CKD-A and CKD-B) using the calculated cut-off values for serum IL-18. CKD-A – patients with serum concentration of IL-18 ≤ 1584.5p g/mL, CKD-B – patients with serum concentration of IL-18 > 1584.5 pg/mL.

CKD-A and CKD-B groups, divided on the basis of the cut-off value assessed for the concentration of IL-18 in the serum, have been compared by the time-to-event analysis (survival analysis). On the basis of the comparison of survival curves (log-rank test) Kaplan-Meier survival analysis revealed that patients classified into CKD-B showed a two-year increased risk for cardiovascular-related death, which served as an endpoint of this study, in comparison to patients forming CKD-A group. The Chi-squared statistics was 62.65 with associated *P*-value of less than 0.00001 showed that the grouping variable (IL-18 ≤ 1584.5 and IL-18 > 1584.5 pg/mL) had a significant influence on survival time among studied patients, see Fig. 3.

The mean survival time, estimated as the area under the survival curve in the interval 0 to *t*_max_, differed between both of the studied groups. In CKD-A it was 23.49 months (95%CI 22.86–24.12) with standard error of estimation (SE) equal to 0.32, unlike the CKD-B with mean survival time of 14.5 months (95%CI 11.15–17.84) with SE 1.71. In CKD-B, the median survival time, considered as the smallest time at which the survival probability drops to 50% or below, was 12 months (95%CI: 8.11–21.12) with SE 1.71. The mean overall survival time in all CKD patients was 21.15 months (95%CI 19.83–22.47) with SE 0.64. The largest difference in the survival rate of these groups was observed in the 23^rd^ month of the duration of the study. In CKD-B the survival proportion was 0.15 with SE = 0.08 while in CKD-A it was equal to 0.93 with SE 0.03. On the other hand, the overall survival proportion in this study was 0.72 with SE 0.05.

The hazard ratio (HR), that compared the hazards in both CKD groups, revealed that patients in CKD-B reached the endpoint (cardiovascular-related death in the 2-year follow-up) 20.63 times more frequently than in CKD-A (95%CI 6.62–64.28).

The relative risk (RR), defined as the ratio of the proportions of cases having a positive outcome in the two CKD groups (CKD-B group serves as an exposed group and CKD-A as a control group) was 12.11 (95%CI 4.62–31.72, *P* < 0.0001). Odds ratio (OR) determined as the ratio of the probability of reaching the endpoint and the probability of failure to achieve this endpoint was 75.08 in CKD-B (95%CI 15.25–369.49, *P* < 0.0001). So, it can be found that both RR and OR were significantly higher in CKD-B than in CKD-A. For details, find it in the Table 4.

These patients who reach the endpoint showed the following results at the enrollment to this study: age > 66 years (52.38% sensitivity (95%CI 29.8–74.3), 75.01% specificity (95%CI 61.6–85.6)), hsCRP > 19.9 mg/L (41.18% sensitivity (95%CI 18.4–67.1), 92.68% specificity (95%CI 80.1–98.5)), ferritin > 278.7 ng/mL (100% sensitivity (95%CI 66.4–100), 40% specificity (95%CI 19.1–63.9)), eGFR ≤ 17 ml/min.1.73 m^2^ (71.43% sensitivity (95%CI 47.8–88.7), 66.07% specificity (95%CI 52.2–78.2)), albumins ≤ 3.9 g/dL (75% sensitivity (95%CI 42.8–94.5), 79.19% specificity (95%CI 57.8–92.9)), CIMT > 1.05 mm (52.38% sensitivity (95%CI 29.8–74.3), 92.86% specificity (95%CI 82.7–99.0)), NT-proBNP > 112.5 fmol/mL (71.43% sensitivity (95%CI 47.8–88.7), 58.93% specificity (95%CI 45–71.9)).

Together with the analysis of the ROC curve, AUC which serves as a summary statistics has been evaluated. If we have taken into consideration the selected variables of a recognized impact on CV risk, such as IL-18, CIMT, NT-proBNP, hsCRP, eGFR, albumins and ferritin, it was disclosed that IL-18 is highly predictive (AUC 0.92, SE 0.05, 95%CI 0.77–0.98); CIMT (AUC 0.84, SE 0.09, 95% CI 0.67–0.94) and albumins (AUC 0.77, SE 0.11, 95%CI 0.55–0.92) are moderately predictive, unlike the less predictive NT-proBNP (AUC 0.66, SE 0.10, 95%CI 0.47–0.81), hsCRP (AUC 0.63, SE 0.10, 95%CI 0.45–0.79), ferritin (AUC 0.63, SE 0.12, 95%CI 0.40–0.82) and eGFR (AUC 0.57, SE 0.11, 95%CI 0.38–0.74).

Next Cox regression that allows analyzing the effect of several risk factors on survival was performed. Using the forward selection method, four models have been conducted. Various factors, including those which have been obtained by the analysis of univariate regression tests have been taken into consideration.

To the first model six parameters (age, BMI, gender, eGFR, statins use and ACEI use) have been entered, however only eGFR was found significantly contributed to the prediction of survival time with *P* < 0.04, HR 0.98, 95%CI 0.96–0.99). The prediction of the model was statistically significant (*P* = 0.026).

To the second model typical cardiovascular-related variables, i.e. age, eGFR, HDL-C, LDL-C and TG, have been included, but only age (*P* = 0.001, HR 1.11, 95%CI 1.04–1.18) and HDL-C (*P* = 0.005, HR 0.94, 95%CI 0.90–0.98) revealed a significant impact on cardiovascular-mortality with P *<* 0.0001. The prediction of the model was highly statistically significant (*P* = 0.0002).

The third model that covered age, albumins, NT-proBNP, CIMT and IL-18, has shown that only two last variables, i.e. CIMT (*P* = 0.003, HR 211.33, 95%CI 11.43–3905.32) and IL-18 (*P* = 0.004, HR 1.29, 95%CI 1.05–1.61) contributed significantly to the cardiovascular-mortality prediction among studied patients. The prediction of the model was highly statistically significant (*P* *<* 0.0001).

Finally, when fifteen covariates, such as: age, BMI, gender, eGFR, HDL-C, LDL-C, TG, albumins, ferritin, hsCRP, NT-proBNP, CIMT, IL-18, statins use, ACEI use, have been taken into account it turned out that in the stepwise regression model (*P* = 0.017), remained IL-18 (*P* *=* 0.0003, HR 14.04, 95%CI 3.69-76.78), so only this covariate contributed to the prediction of survival time in this model. The prediction of this model was highly statistically significant (*P* = 0.0003).

##### Factors influencing serum IL-18

In correlative analysis, serum IL-18 concentration was found to be positively correlated with CIMT (r = 0.73, *P* = 0.00), hsCRP (r = 0.54, *P* = 0.00), PTH (r = 0.56, *P* = 0.00), PO_4_^3-^ (r = 0.55, *P* = 0.00), ferritin (r = 0.66, *P* = 0.00), NT-proBNP (r = 0.45, *P* = 0.00), ACEI use (r = 0.46, *P* = 0.04) and inversely associated with eGFR (r = −0.75, *P* = 0.00), HGB (r = −0.61, *P* = 0.00), HDL-C (r = −0.35, *P* = 0.00), albumins (r = −0.25, *P* = 0.03) and statins use (r = −0.46, *P* = 0.02).

There were no statistically significant correlations between IL-18 and selected parameters such as: age, BMI, TG, TC, LDL-C, Ca total, beta-blockers and NSAID.

Next, to identify parameters that may determined IL-18 level in the serum, multiple linear step-wise regression analysis with IL-18 as a dependent variable, was performed. When significant factors, like eGFR, HDL-C, CIMT, hsCRP, ferritin, PO_4_^3-^, albumins, PTH, NT-proBNP, statins use and ACEI use chosen by the univariate regression analyses were entered to this analysis, it allowed for determination of four variables, such as CIMT, NT-proBNP, albumin level and hsCRP as independent factors for IL-18 serum variability. They accounted for 89.5% of the variance in the level of serum IL-18. The strongest effect on serum concentration of IL-18 showed CIMT (the largest standardized BETA-coefficient, independent of the sign). The formulated final multiple regression model has been presented in Table 5.

To estimate the robustness of an IL-18 cut-off the sensitivity analysis has been performed using the following parameters: PO_4_^3-^ (1.87), CIMT (1.77), age (1.6), eGFR (1.25), hsCRP (1.00), sex (0.99), HDL-C (0.98), TG (0.95), ferritin (0.91), HGB (0.89), BMI (0.82), NT-proBNP (0.49), LDL-C (0.45), total cholesterol (0.35). Assuming that, if the estimated value (in parenthesis) is 1 or less, the influence of the variable can be omitted entirely, only the first four of the mentioned variables (i.e. PO_4_^3-^, CIMT, age, eGFR) can be consider as important predictors of IL-18, although their influence is rather very weak.

## Discussion

In the current study, using systems and clinical approach, the important role of IL-18 in the signaling pathways of atherosclerosis, that is the leading cause of morbidity and mortality worldwide, has been confirmed. In addition, it has been demonstrated for the first time that in the 2-year follow-up the concentration of IL-18 in the serum may reflect the risk of cardiovascular-related death among patients with CKD and AMI in the preceding year. Patients characterized by high serum concentrations of IL-18 (above the cut-off value) showed worse survival rate compared to the patients with lower concentration of this cytokine. It should be emphasized that these results related to the patients without diabetes mellitus. On the other hand, it needs to be stressed that this calculated cut-off value was higher than usually reported in the literature. The reasons for this may be several. Firstly, it should be underlined that all studied patients showed higher levels of serum IL-18 as compared with the control group (HV). As the kidneys are probably the major sites of cytokines elimination, the primary factor which might affect these results, was CKD diagnosis, responsible for the decreased renal clearances of IL-18. Of note, IL-18 is a middle-molecule and protein-bound uremic toxin, which is difficult to remove by any of the currently available dialytic strategies^15^, thus the observed accumulation of IL-18 in dialyzed patients. Secondly, the activation of the monocyte/macrophage network found during dialysis session produces multiple inflammatory cytokines, what may also explain the increase of IL-18 in the serum. Thirdly, IL-18 has been recently implicated in atherosclerotic plaque instability, what in studied patients may also be the cause of their elevated levels of IL-18. In addition, besides implications for plaque instability, IL-18 has been recently discovered to be involved in myocardial ischemia-reperfusion injury. Several potential mechanisms leading to abnormal remodeling and heart failure are being considered^16^. In this light, the history of AMI and the emergence of the next in studied patients can also be a factor causing an IL-18 increase. Mallat *et al.*^16^ demonstrated that IL-18 is up-regulated in the myocardium of patients suffering from decompensated heart failure of ischemic or non-ischemic origin compared with patients without heart failure. This increase was found to induce apoptosis in activated T cells, leading to persistent myocardial damage^17^. Other researchers confirmed this and proposed to use IL-18 as a marker of the acute inflammatory process and an index of myocardial necrosis^18^.

In view of all mentioned aspects, it should not be surprising that in our study we used such a high cut-off point for the tested cytokine. High sensitivity (81%) and specificity (96.4%) of this cut-off point was reflected in the results that have been generated on its basis. Namely, the two groups (groups CKD-A and CKD-B) formed in the relation to the cut-off point, had completely different cardiovascular risk and prognosis in a 2-year follow-up. Patients classified to the group with serum IL-18 that exceed the cut-off point revealed the definitely increased risk (HR 20.63, OR 75.08) for 2-year cardiovascular mortality in comparison to the CKD-A group with serum IL-18 that was lower than/equal to the cut-off point. The sensitivity analysis of this cut-off point revealed that only few parameters, such as PO_4_^3^, CIMT, age, and eGFR can be found to be important predictors, although their influence is rather very weak. Of course we realize that the IL-18 cut-off has to be evaluated in a completely independent population for confirmation. Such a study will be prepared in the near future.

Comparing the different variables of a widely recognized impact on cardiovascular risk we have shown IL-18 advantage over other parameters such as CIMT and albumins (moderately predictive), and NT-proBNP, hsCRP, eGFR and albumins (less predictive). Moreover, IL-18 has proved to be also the best predictor for two-year cardiovascular mortality in stepwise regression analysis. In combination with age, BMI, gender, eGFR, HDL-C, LDL-C, TG, albumins, ferritin, hsCRP, NT-proBNP, CIMT, statins use, ACEI use it turned out to be the only variable with a statistically significant impact on the cardiovascular mortality.

Next, the linear stepwise regression analysis has allowed to find four parameters, like CIMT, NT-proBNP, albumins and hsCRP, which are the best predictors for IL-18 levels, as they accounted for 89.5% of the variance in the level of IL-18 in the studied patients (*F* = 21.3, *P* < 0.00007). The strongest of these parameters proven to be CIMT and its association with IL-18 appears to be indisputable. In turn, the relations between NT-proBNP and IL-18 are not so obvious. Mallat *et al.*^16^, found that there is no correlation between IL-18 and BMP levels in human heart failure, suggesting that they reflect different physiological mechanisms. On the other hand, DiSomma *et al.*^19^ discovered that IL-18 in view of its ability to exert inflammatory, hypertrophic and pro-fibrotic activities stimulates BNP synthesis by cardiomyocytes *in vitro* studies and correlates with BNP in non-overloaded acute heart failure patients, and in patient with heart failure, diabetes mellitus, and CAD. Moreover, Cordero-Reyes *et al.*^20^, in their pre-clinical studies found positive correlations between BNP and IL-18 levels in patients with acute decompensated heart failure, suggesting a potential role of B-cells in their outcome. In our study we evaluated NT-proBNP and we noticed a strong positive correlation between IL-18 and this parameter, although it was not the best predictor of cardiovascular mortality and decisively inferior to IL-18. Another parameter which turned out to significantly affect the variability of IL-18 was albumins level. Although albumins have been discovered to be a strong predictor of mortality in ESRD^21^, correlations between albumins and pro-inflammatory cytokines levels are still analyzed^9^,^11^. Our data showed that there is significant reverse relationship between IL-18 and albumins. The last of the parameters which were put in the formula describing the variability of IL-18 was hsCRP, a sensitive marker of inflammation, tissue damage, and infection reflecting the degree of underlying inflammatory response and being a useful measure of immune injury to tissues. Yamaoka-Tojo *et al.*^22^, showed that CRP may contribute to the endothelial inflammation in acute coronary syndrome by activation of the IL-18 system, which may amplify the inflammatory cascade in tissue injury in addition to initiating endothelial damage and atherosclerosis. However, in our study the usefulness of the use of hsCRP as an indicator of cardiovascular death was lower as compared with IL-18.

It is evident that the concentration of IL-18 in serum reflects severity of the chronic inflammation, function of the kidneys, and the increase in CIMT accompanying CKD. It is difficult to ascertain if IL-18 is a good indicator of severity of atherosclerosis, but it seems to be a good indicator of its clinical complications among patient suffering from CKD, without diabetes mellitus, and with AMI in the year prior to the enrolment. Of course, we are aware that such a short period of follow-up in our study is certainly a limit and it is difficult to formulate far-reaching conclusions. There are also other limitations for the current study. One of them is the selection bias of the study group. We excluded patients if one or more of the following conditions were present: diabetes mellitus, kidney transplants, albuminuria, current infection, immunosuppression, malignant tumors ≤ 10 years, smoking and/or alcohol abuse ≤ 10 years, leading to a selected group of patients which may contribute to low generalizability/external validity of the results. Secondly, we used a single time point measurement, which does not guarantee the average levels in our patients. On-going infection or inflammatory diseases could lead to transiently higher IL-18 levels. In addition, if renal function declined during the observation period, an increase in IL-18 levels can be expected. Thus, rapid loss of renal function might after 2 years results in increased levels in patients with initial low IL-18. This aspect may weaken the predictive value of IL-18. Thirdly, this study included substantial number of patients on medications, requiring larger studies to separate the effects of such medications. Although we noted the existence of some relationships between the concentrations of IL-18 and statins, and ACEI is not possible in this study, because of its structure, to examine the impact of these drugs on serum IL-18 and check their influence on the obtained results. However, new studies will be conducted to explain this issue. Fourthly, it should be also underlined that total IL-18 levels, used in our study, may not reflect the biologically active free IL-18 because several natural inhibiting binding proteins exist, which are reactive to inflammatory stress and were found to modulate atherosclerotic lesion development in apoE^−/−^mice^23^,^24^. Finally, it should also be remembered that IL-18 levels, in both healthy and diseased individuals, are determined in part by genetic variation within IL-18^25^.

Despite limitations, it should be emphasized that IL-18, due to its pleiotropic properties seem to be important indicator of cardiovascular-related death in non-diabetic, hypertensive patients with CKD, especially those under long-term HD. The principal finding in this study is that IL-18 serum concentrations equal to or lower than the evaluated cut-off values may protect non-diabetic patients with varying stages of CKD and history of AMI in the previous year, against cardiovascular death in 2-year follow-up. Moreover, in this study, we have shown superiority of the use of IL-18 on hsCRP, CIMT, eGFR, albumins, ferritin and NT-proBNP in the prediction of cardiovascular mortality in the studied CKD patients. The effects of circulating IL-18 on long-term clinical outcomes were not part of the protocol, so all of the observed relationships did not extend beyond the two-year clinical outcome endpoint.

## Methods

### Ethics Statement

The study was carried out in accordance with the Declaration of Helsinki of the World Medical Association and approved by the Ethical Committee of Poznan University of Medical Sciences. All included study participants fulfilled criteria and completed the study. They were fully informed about the study and all of them gave written informed consent before their examination.

### Study design

This study enrolled a total of 197 consecutive patients, aged 45–81 years, admitted to the Department of Nephrology, Transplantology and Internal Medicine at Poznan University of Medical Sciences or treated in the Department of Hemodialysis at Poznan University of Medical Sciences or in outpatient clinic, in the years 2008-2011. They were assessed for eligibility and five of them were discharged from the study due to the mental health difficulties. All qualified persons underwent a careful interview and a clinical examination with an evaluation of patients’ history based on hospital and outpatient records. They were assigned to one of the CKD groups, according to the severity of their kidney disease. Identification and staging of CKD was based on measurement of estimated glomerular filtration rate (eGFR) and albuminuria level.

On the basis of the clinical interview and clinical examination it was found that 192 patients fulfilled our inclusion criteria. They were as follows: CKD (CKD has been defined as either kidney damage or reduced estimated GFR to < 60 mL/min/1.73 m^2^ sustained for at least 3 months, irrespective of the type of kidney disease), clinically stable state, history of AMI treated with percutaneous transluminal coronary angioplasty (PTCA) in the past year prior to enrolment, no diabetes mellitus, hypertension (diagnosed when a patient received antihypertensive drugs or at the time of the study had a repeatedly elevated blood pressure exceeding 140 over 90 mmHg). On the other hand, the exclusion criteria were the following: albuminuria - defined as urinary albumin to creatinine ratio ≥30 mg/g, current or recent (<1 month) active acute infection, immunosuppressive treatment, kidney transplantation, abnormal liver function, malignant tumors in the past 5 years, smoking and/or alcohol abuse in the past 5 years. Criteria associated with albuminuria come from the fact that some of participants of this study created a significant part of the groups that have been analyzed in our previous proteomic studies^4^,^26^.

**Figure 4:**
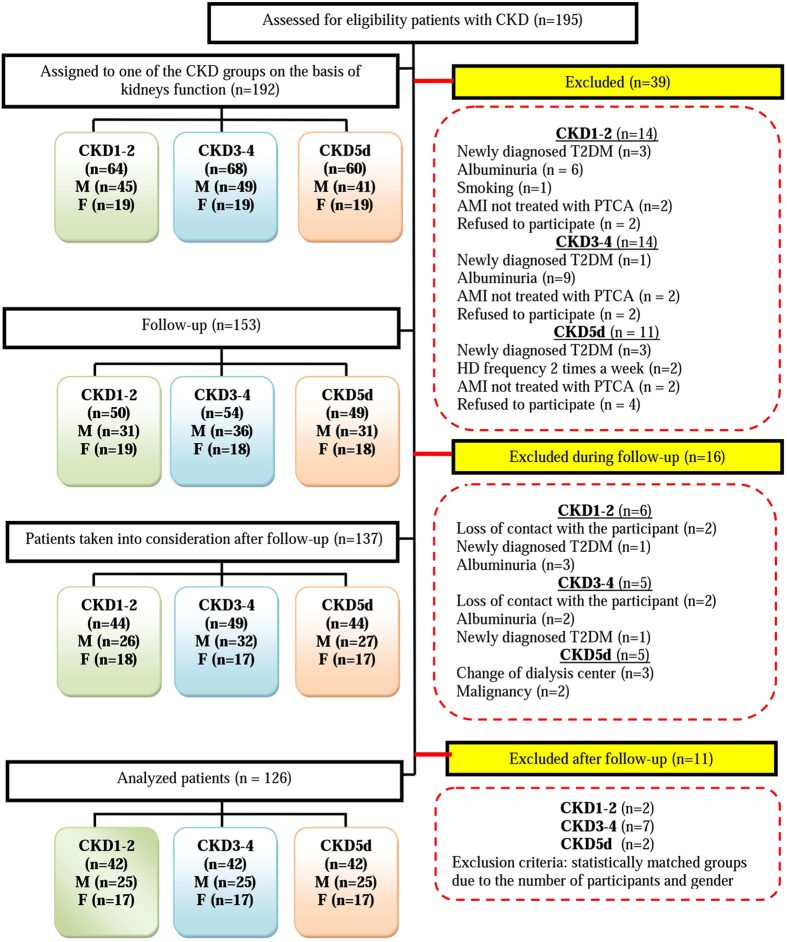
The flow of participants from enrolment to analysis.

Moreover, after obtaining the additional data from the patients, followed by the analyses of their results of basic laboratory tests, 39 of them were excluded from the study. The details of exclusion are presented in Fig. 4. Hence, to the 2-year follow-up 153 patients were qualified, of whom 16 were excluded during this time due several reasons, i.e. loss of contact (4 patients), change of dialysis center (3 patients), detection of albuminuria (5 patients), newly diagnosed diabetes mellitus type 2 (T2DM) (2 patients) and malignancy (2 patients).

Finally, after a 2-year follow-up, 11 participants were excluded from the study after statistical adjustment of data, to get the groups of equal cardinality and not differing in gender distribution.

Hence, 126 CKD patients (51 women and 75 men) forming three groups were taken into account in the analyses. The first group (CKD1-2) was formed by 42 patients (stages 1^st^ and 2^nd^ of CKD), characterized by eGFR ≥ 90–60 ml/min/1.73 m^2^. The second group (CKD3-4) consisted of 42 patients (stages 3^rd^ and 4^th^ of CKD) with eGFR = 15–59 ml/min/1.73 m^2^. To the third group (CKD5d) 42 hemodialysed patients were classified with a range of hemodialysis treatment of 7–156 months (mean hemodialysis vintage 37.07 ± 34.56 months). They were dialyzed using low-flux polysulphone membrane, 3 times and a week with prescriptions of 4.5–5.5 hours/session and mean dialysis adequacy Kt/V 1.25 ± 0.28. Diagram presenting the flow of participants and cardinality at each stage of the study from enrollment, 2-year follow-up and analysis, has been shown in Fig. 4.

The causes of CKD were various: chronic glomerulonephritis (44 patients), hypertensive nephrosclerosis (32 patients), chronic interstitial nephritis (26 patients), polycystic kidney disease (9 patients) and unknown causes (15 patients). However, the number of cases with these diagnoses unfolded almost evenly in all CKD groups. Patients received drugs, i.e. aspirin, beta-blockers, statins, ACE inhibitors, in standard doses according to the recommendation. Moreover, after hemodialysis session patients enrolled to the CKD5d group have been supplemented intravenously, according to NKF KDOQI™ guidelines with the recombinant human erythropoietin (39 patients) and with undiluted iron sucrose-ferric hydroxide saccharin (32 patients), prescribed in slow injection given directly into the dialysis line.

When CKD patients formed the final study groups, a reference group of 52 healthy volunteers (HV) was created. It comprised mainly of blood donors, matched for age and gender to CKD groups, without albuminuria and AMI in the past, with proper kidney function considered when eGFR > 60 mL/min/1.73 m^2^.

### Data collection

Blood samples have been obtained from fasting participants. Moreover, from hemodialysed patients’ blood samples have been taken before the onset of the second hemodialysis session of the week. All samples were stored and processed in an identical fashion. All of assessed laboratory parameters were performed using standard methods, according to the manufacturer’s instruction.

### Follow-up CV-related deaths

Patients were followed for 2 years. The primary endpoint was fatal AMI, fatal acute ischemic stroke or any sudden or unexpected death unless proven to be non-CV at autopsy.

### The methodology used for estimating the analyzed parameters

Serum IL-18 concentration was determined by commercially available immunoenzymatic method (Colorimetric Sandwich ELISA, Quantikine Human IL-18 R&D Inc., USA) with detection limit bellow 12.5 pg/ml.

The complete blood count, glucose (Glu), total cholesterol (TC), low-density lipoprotein cholesterol (LDL-C), high-density lipoprotein cholesterol (HDL-C), triglycerides (TG), creatinine, Ca total, PO_4_^3-^, intact parathormon (iPTH), total protein (TP), albumins were assessed by routine techniques using automated analyzers Sysmex K-4500 (ICN) and Cobas Integra 400 (Roche), from fasting blood samples.

High-sensitivity C-reactive protein (hsCRP) [mg/l] was measured using a wide-range latex-enhanced immunoturbidimetric assay on an ADVIA 2400 analyzer (Siemens Healthcare Diagnostics, U.K.).

Body mass index (BMI) [kg/m^2^] was calculated by dividing a person's weight (post-dialysis weight in CKD5d) in kilograms by the square of their height in meters.

Carotid intima-media thickness (CIMT) was measured by The Accuson CV 70 system (Simens) with a 10 MHZ transducer. Two longitudinal projections were assessed (anterolateral and posterolateral). The distal 1 cm of the common carotid artery just proximal to the bulb was measured by means of a computer analysis system (Medical Imaging Applications, LLC).

### Statistical approach

Analyses have been performed on the input data, mean values or ranks depending on the applied tests. Data were expressed as mean ± standard deviation (SD), and as proportions for categorical data. Normality of variable distribution has been analyzed using Shapiro-Wilk W-test. The comparisons of the normally distributed variables between two studied groups have been performed using the unpaired Student *t*-test. For multiple comparisons of the normally distributed variables the one-way analysis of variance (ANOVA) test has been performed. The differences between two not normally distributed continuous variables from two independent groups were calculated by nonparametric analyses with the Mann-Whitney U tests, but for the multiple comparisons the Kruskal-Wallis test was used, followed by multiple comparisons of mean ranks for all groups. For categorical comparisons chi square test was performed.

The risk for CV-related death was calculated using the Kaplan-Meier method. The cut-off point of circulating IL-18 concentration in the serum was estimated from the receiver operating characteristic (ROC) curve analysis. Together with the analysis of the ROC curve, the area under the ROC curve (AUC) has been evaluated. On the basis of ROC analysis performed for serum concentration of circulating IL-18, CKD groups have been stratified into two groups, i.e. CKD-A and CKD-B, characterized by the serum IL-18 concentration of less than or equal to the cut-off and higher than the cut-off point, respectively. The survival distributions of these two groups were analyzed using the log-rank test. The uni- and multivariate Cox regression analyses have been used to find the risk factors for CV-related mortality. Forward Cox proportional hazard regression models for censored survival data have been used for assessing the associations between baseline factors and CV-related death incidents, independent of other predictors of survival. Moreover, the sensitivity analysis for estimating the robustness of an IL-18 cut-off has been conducted.

Linear regression analysis employed Pearson's or Spearman's coefficients as appropriate. Multiple linear step-wise regression analysis with IL-18 as a dependent variable was performed to identify parameters which determined its concentration in the serum. Significant independent variables were ordered according to their standardized effect, defined as regression coefficient/standard error of the regression.

Statistical significance was set at *P* < 0.05 for all tests. Data given were analyzed using STATISTICA version 10.0 software (Stat Soft Inc., USA) and MedCalc Statistical Software version 15.2.1 (MedCalc Software bvba, Ostend, Belgium; http://www.medcalc.org; 2015).

### Systems approach

Petri nets are mathematical objects which have a structure of a directed bipartite graph, what means that they are composed of vertices of two types called places and transitions connected by arcs (i.e. directed edges). In such a net only vertices of various types can be directly connected (i.e. there are no arcs between two places nor between two transitions). Moreover, each arc is labeled by a weight being a positive integer number. The directed bipartite graphs determine the structure of Petri nets but one of the fundamental properties of the nets is a dynamics. This dynamics is associated with tokens flowing through the net from one place to another via transitions. When a Petri net is a model of a biological system places correspond to its passive components, like chemical compounds, while transitions are counterparts of active components of the system, like chemical reactions. Tokens residing in places correspond to quantities of the passive components.

The flow of tokens is governed by a simple transition firing rule according to which a transition is enabled if in every place being its direct predecessor (called its pre-place) the number of tokens is equal to or greater than the weight of an arc connecting the place with the transition. An enabled transition can be fired what means that tokens can flow from all of its pre-places to its direct successors (called its post-places). The number of flowing tokens is equal to the weight of a given arc. There are two exceptions to this rule, i.e. a transition without pre-places is always enabled while a transition without post-places when fired does not produce tokens. The flow of tokens corresponds to a flow of substances, information etc. through the modeled system.

Petri nets have very intuitive graphical representation, where places are shown as circles, transitions as rectangles, arcs as arrows, tokens as dots or numbers within places and weights as numbers associated with arcs (if a weight is equal to one it is usually not explicitly shown in this representation). Despite that the graphical representation is very useful and helps to understand the structure and behavior of the net it is not well-suited for the formal analysis of its properties. For this purpose another representation called an incidence matrix is used. Such matrix *A* is composed of *n* rows and *m* columns, where *n* is the number of places while *m* is the number of transitions. A number in *i*-th row and *j*-th column is equal to a difference between the number of tokens residing in *i*-th place before and after firing *j*-th transition^27^,^28^.

Petri nets have many interesting properties but in the context of biological systems especially important are their t- and p-invariants. A t-invariant is vector *x* of integer numbers being a solution to the equation *A*·*x* = 0. Similarly, a p-invariant is a vector *y* which is a solution of the equation *y*·*A* = 0. t-invariants corresponds to sets of transitions whose firing a proper number of times (which for the j-th transition is equal to the j-th invariant entry) does not change the distribution of tokens over places (i.e. the state of the system). In other words, while transitions correspond to elementary sub-processes, t-invariants correspond to more complex sub-processes of the system which have this important property. On the other hand, p-invariants correspond to sets of places for which the number of tokens remains constant^28^,^29^.

An analysis of similarities between t-invariants may lead to finding some dependencies between the corresponding biological sub-processes and discovering previously unknown properties of the modeled system. These similarities can be analyzed using clustering methods^30^. However, not only the analysis of similarities between t-invariants may lead to interesting conclusions but also the composition of individual invariants is a source of knowledge about the interactions among elementary processes of the system. From these interactions follows many properties of the investigated biological phenomenon, so the analysis of them may contribute to deeper understanding of its nature.

Moreover, in the modeled system there can be indicated groups of transitions which are elements of exactly the same t-invariants (formally, they are elements of supports of the same t-invariants, see^28^). These groups are called Maximum Common Transition sets (MCT-sets) and correspond to some functional modules of the biological system^30^.

## Additional Information

**How to cite this article**: Formanowicz, D. *et al.* Usefulness of serum interleukin-18 in predicting cardiovascular mortality in patients with chronic kidney disease – systems and clinical approach. *Sci. Rep.* **5**, 18332; doi: 10.1038/srep18332 (2015).

## Figures and Tables

**Table 1:** List of non-trivial MCT-sets.

MCT	No. of the contained transitions	Biological meaning
1	15	MyD88-dependent signaling pathway
2	7	induction of apoptosis influenced by caspases 3 and 8 that are situated at pivotal junctions in apoptosis pathways
3	4	activation of the innate immune system through TLR signaling pathways regulated by TIR domain-containing adaptors, such as MyD88
4	4	JAK-STAT signaling pathway stimulated by INF-γ and its impact on the regulation of iNOS expression
5	3	formation of an active TNFR1 signaling complex
6	3	influence of NO on cardiovascular system
7	3	transformation of monocytes into activated macrophages
8	3	activation of a silencer of TNFR1 signaling pathway
9	2	pro-IL-18 signaling pathway
10	2	activation of macrophages by the classical pathway
11	2	SODD signaling pathway

Abbreviations: INF-γ – interferon gamma; iNOS – inducible isoform nitric oxide synthase; JAK/STAT – Janus kinase/signal transducers and activators of transcription; MyD88 – myeloid differentiation primary response 88; NO – nitric oxide; SODD – silencer of death domains protein; TIR - Toll/interleukin-1 receptor; TLR – Toll-like receptor; TNFR1 – tumor necrosis factor receptor 1.

**Table 2:** Clinical and biochemical characteristics of the analyzed groups.

	study groups				
	CKD (n = 126)			HV (n = 52)	P*
	CKD1–2 (n = 42)	CKD3–4 (n = 42)	CKD5d (n = 42)		
age [years]	60.41 ± 6.42	63.61 ± 11.91	62.62 ± 9.92	61.91 ± 9.71	**0.47**
gender	25 males 17 female	25 males 17 female	25 males 17 female	31 males 21 female	**0.63**
BMI [kg/m^2^]	28.21 ± 4.51	26.32 ± 2.11	24.72 ± 3.71	24.61 ± 2.11	**0.00**
systolic blood pressure [mmHg]	124.96 ± 13.85	139.62 ± 8.25	133.32 ± 27.77	123.96 ± 11.71	**0.04**
diastolic blood pressure [mmHg]	75.62 ± 10.12	85.36 ± 75.29	77.26 ± 17.20	67.48 ± 13.63	**0.06**
Treatment at discharge that may have a considerable influence on the obtained results [n (%)]					
ACEI	34 (80.11%)	21 (50.0%)	14 (33.33%)	0 (0%)	**0.00**
beta-blockers	24 (57.14%)	25 (59.52%)	26 (61.91%)	0 (0%)	**0.00**
statins	40 (95.23%)	33 (78.51%)	34 (80.11%)	0 (0%)	**0.00**
NSAID	40 (95.23%)	33 (78.51%)	16 (23.81%)	0 (0%)	**0.00**
Laboratory tests					
eGFR [ml/min/1.73 m^2^]	84.25 ± 20.19	28.74 ± 15.68	5.33 ± 6.98	119.42 ± 25.93	**0.00**
RBC [1012/l]	4.51 ± 0.82	3.91 ± 0.82	3.63 ± 0.53	4.72 ± 0.41	**0.00**
HGB [g/dl]	13.21 ± 3.12	11.61 ± 1.31	11.41 ± 1.51	14.02 ± 0.92	**0.00**
HCT [l/l]	42.03 ± 5.81	36.43 ± 4.42	31.31 ± 4.62	43.12 ± 5.13	**0.00**
WBC [109/l]	7.31 ± 2.11	7.02 ± 1.81	6.62 ± 1.83	5.42 ± 0.91	**0.00**
glucose [mg/dl]	83.51 ± 9.53	81.81 ± 9.22	81.01 ± 9.53	79.12 ± 10.11	**0.63**
iron [μg/dl]	89.21 ± 43.13	74.26 ± 27.65	76.31 ± 28.32	113.41 ± 23.82	**0.00**
ferritin [ng/ml]	207.61 ± 111.41	321.71 ± 212.41	1084.91 ± 740.51	209.12 ± 110.91	**0.00**
Ca total [mg/dL]	7.11 ± 2.71	8.01 ± 0.41	9.61 ± 1.42	8.61 ± 0.21	0.13
PO43- [mg/dl]	3.68 ± 1.81	3.74 ± 1.52	7.01 ± 3.56	3.4 ± 0.7	**0.00**
iPTH [pg/ml]	89.41 ± 21.12	197.42 ± 138.62	320.82 ± 207.41	38.11 ± 6.82	**0.00**
total protein (TP) [g/dL]	6.61 ± 1.72	5.52 ± 1.32	6.22 ± 0.69	7.21 ± 0.43	**0.00**
albumins [g/dL]	4.0 ± 0.96	4.13 ± 1.84	3.82 ± 0.69	4.24 ± 0.64	0.07
total cholesterol (TC) [mg/dl]	217.02 ± 53.61	182.31 ± 28.12	178.31 ± 48.92	188.71 ± 27.03	**0.00**
HDL-C [mg/dl]	55.01 ± 13.31	58.41 ± 1.82	46.61 ± 23.21	70.61 ± 6.71	**0.00**
LDL-C [mg/dl]	168.61 ± 48.22	120.61 ± 17.11	105.93 ± 50.71	93.94 ± 30.21	**0.00**
TG [mg/dl]	175.11 ± 41.42	126.61 ± 14.82	151.3 ± 50.81	120.71 ± 37.82	**0.01**
hsCRP [mg/l]	9.91 ± 8.21	10.71 ± 11.23	12.62 ± 11.93	1.12 ± 0.43	**0.00**
CIMT [mm] (min-max)	0.71 ± 0.21 (0.53–1.16)	0.82 ± 0.21 (0.50–1.26)	0.91 ± 0.41 (0.43–1.48)	0.45 ± 0.28 (0.22–0.72)	**0.00**
NT-proBNP [fmol/mL]	48.46 ± 36.95	131.33 ± 103.18	224.05 ± 102.84	9.35 ± 3.92	**0.00**
IL-18 [pg/mL]	818.10 ± 333.81	1207.46 ± 659.96	1454.07 ± 762.65	185.75 ± 94.18	**0.00**

^*^Comparisons between studied groups were assessed by the ANOVA rang Kruskal-Wallis tests used for nonparametric comparisons, whereas for categorical variables chi square test was performed.

Continuous variables are presented as mean values ± standard deviation; categorical variables are presented as percentage of the number of persons in a given group. Bold font highlights statistically significant differences between studied groups with *P* < 0.05, which was considered as statistically significant. ACEI – angiotensin-converting enzyme inhibitors; NSAID – non-steroidal anti-inflammatory drugs.

**Table 3:** Clinical and biochemical characteristics of CKD groups divided, into two groups: CKD-A and CKD-B, on the basis of the calculated cut-off values for IL-18 serum concentration.

	Groups of patients		P*
	CKD (n = 126)		
	CKD-A (n = 91) IL-18 ≤ 1584.5 pg/mL	CKD-B (n = 35) IL-18 > 1584.5 pg/mL	
age [years]	61.17 ± 9.98	65.65 ± 7.75	0.06
gender	50 males 41 female	25 males 10 female	0.01
BMI [kg/m^2^]	26.34 ± 3.17	26.41 ± 3.12	0.91
systolic blood pressure [mmHg]	131.1 ± 18.41	129.59 ± 31.55	0.48
diastolic blood pressure [mmHg]	78.67 ± 11.65	72.39 ± 18.46	0.29
CKD stages [n]			
CKD1-2	40	2	
CKD3-4	28	14	**0.00**
CKD5d	23	19	
HD vintage in CKD5 group [months]	9.56 ± 25.39	22.8 ± 29.43	**0.01**
Kt/Vin CKD5 group	1.24 ± 0.32	1.27 ± 0.21	0.82
Treatment at discharge that may have a considerable influence on the obtained results [n (%)]			
ACEI	51 (56.04%)	18 (51.42%)	0.19
beta-blockers	54 (59.34%)	21 (60.00%)	0.43
statins	76 (83.51%)	31 (88.57%)	0.39
NSAID	62 (68.13%)	27 (77.14%)	0.19
Laboratory tests			
eGFR [ml/min/1.73 m^2^]	43.03 ± 36.54	18.65 ± 19.06	**0.01**
RBC [1012/l]	3.98 ± 0.77	3.91 ± 0.71	0.35
HGB [g/dl]	12.14 ± 2.21	11.61 ± 1.81	0.30
HCT [l/l]	37.80 ± 6.01	35.33 ± 5.01	0.22
WBC [109/l]	6.61 ± 2.06	6.63 ± 2.04	0.80
glucose [mg/dl]	90.97 ± 10.77	89.27 ± 12.06	0.75
iron [μg/dl]	80.12 ± 30.41	69.91 ± 20.92	0.71
ferritin [ng/ml]	691.87 ± 529.30	1229.12 ± 1169.54	0.29
Ca total [mg/dl]	8.92 ± 4.01	9.21 ± 0.81	0.18
PO43- [mg/dl]	6.70 ± 4.21	6.19 ± 1.86	0.51
iPTH [pg/ml]	320.46 ± 196.34	288.50 ± 234.62	0.76
TP [g/dL]	6.68 ± 1.06	6.39 ± 0.57	0.17
albumins [g/dL]	4.34 ± 1.34	3.65 ± 0.51	0.02
TC [mg/dl]	198.26 ± 54.38	178.93 ± 45.53	0.14
HDL-C [mg/dl]	53.84 ± 23.97	41.51 ± 14.98	0.16
LDL-C [mg/dl]	131.61 ± 55.31	119.18 ± 50.17	0.10
TG [mg/dl]	155.17 ± 66.23	120.85 ± 29.39	0.07
hsCRP [mg/l]	10.03 ± 9.70	15.27 ± 13.29	0.15
CIMT [mm] (min-max)	0.75 ± 0.21 (0.43–1.27)	0.96 ± 0.33 (0.45–1.48)	**0.02**
NT-proBNP [fmol/mL]	124.19 ± 111.82	179.77 ± 109.93	**0.03**
IL-18 [pg/mL]	837.48 ± 303.85	2147.075 ± 422.91	**0.00**
CKD stages [IL-18 pg/mL]			
CKD1-2	745.93 ± 244.95	1447.01 ± 215.78	**0.00**
CKD3-4	927.79 ± 409.21	1969.21 ± 678.47	**0.00**
CKD5d	953.12 ± 383.47	2182.72 ± 553.30	**0.00**

^*^Comparisons between both studied groups assessed by the Mann - Whitney U tests or unpaired Student t-test, depending on the normality of the distribution of variables. For categorical variables chi square test was performed.

Continuous variables are presented as mean values ± standard deviation; categorical variables are presented as percentage of the number of persons in a given group. Bold font highlights statistically significant differences between studied groups with P < 0.05, which was considered as statistically significant.

**Table 4:** Relative risk and odds ratio in CKD-A and CKD-B groups.

	Outcome		RR (95%CI)	OR (95%CI)
	patients who did not achieve the endpoint* (n = 93)	patients who achieved the endpoint*(n = 33)		
CKD-A IL-18 ≤ 1584.5 pg/mL (n = 91)	86	5	0.08 (0.03–0.21) P < 0.0001	0.01 (0.002–0.06) P < 0.0001
CKD-B IL-18 > 1584.5 pg/mL (n = 35)	7	28	12.11 (4.62–31.72) P < 0.0001	75.08 (15.25–369.49) P < 0.0001

Abbreviations: *endpoint = cardiovascular-related death in the 2-year follow-up; RR – relative risk; OR – odds ratio.

**Table 5:** Regression analysis with IL-18 in serum as a dependent variable#.

Coefficients^a^						
independent variable	standardized coefficients		unstandardized coefficients		t	*P*
	BETA	std. error of BETA	B	std. error of B		
intercept			−1.03	0.32	−3.17	0.00
CIMT	0.75	0.11	0.03	0.00	6.42	0.00
NT-proBNP	0.45	0.10	0.63	0.14	4.22	0.00
albumins	−0.51	0.11	−0.005	0.00	−4.53	0.00
hsCRP	0.32	0.10	0.01	0.01	3.01	0.01

^a^dependent variable: IL-18;

^#^the coefficient of determination *R*_^2^_ = 89.5%, the ratio of the model mean square to the error mean square *F* = 21.3, standard error of estimation SE = 0.18, *P* < 0.00007.
